# Association of CYP1A1 MspI polymorphism in the esophageal cancer risk: a meta-analysis in the Chinese population

**DOI:** 10.1186/s40001-015-0135-3

**Published:** 2015-03-30

**Authors:** Hui Zheng, Yun Zhao

**Affiliations:** Nursing Department, Tai’an Tumor Hospital, Tai’an City, No. 262 Taidong Road, Shandong Province 271000 China; Pathology Department, Tai’an Tumor Hospital, Tai’an City, No. 262 Taidong Road, Shandong Province 271000 China

**Keywords:** Meta-analysis, CYP1A1 MspI, Polymorphism, Esophageal cancer, Chinese

## Abstract

**Background:**

Although many epidemiologic studies have investigated the CYP1A1 MspI gene polymorphisms and their associations with esophageal cancer (EC), definite conclusions cannot be drawn. To clarify the effects of CYP1A1 MspI polymorphisms on the risk of EC, a meta-analysis was performed in Chinese population.

**Methods:**

Related studies were identified from PubMed, Springer Link, Ovid, Chinese Wanfang Data Knowledge Service Platform, Chinese National Knowledge Infrastructure (CNKI), and Chinese Biology Medicine (CBM) till October 2014. Pooled ORs and 95% CIs were used to assess the strength of the associations.

**Results:**

A total of 13 studies including 1,519 EC cases and 1,962 controls were involved in this meta-analysis. Overall, significant association was found between CYP1A1 MspI polymorphism and EC risk when all studies in the Chinese population pooled into this meta-analysis (C *vs*. T: OR = 1.25, 95% CI = 1.04 to 1.51; CC + CT *vs*. TT: OR = 1.35, 95% CI = 1.06 to 1.72; CC *vs*. TT + CT: OR = 1.35, 95% CI = 1.03 to 1.76). When we performed stratified analyses by geographical locations, histopathology type, and source of control, significantly increased risks were found in North China (C *vs*. T: OR = 1.38, 95% CI = 1.12 to 1.70; CC *vs*. TT: OR = 1.72, 95% CI = 1.16 to 2.56; CC + CT *vs*. TT: OR = 1.52, 95% CI = 1.14 to 2.02; CC *vs*. TT + CT: OR = 1.55, 95% CI = 1.17 to 2.06), in the population-based studies (C *vs*. T: OR = 1.22, 95% CI = 1.05 to 1.42; CC *vs*. TT: OR = 1.38, 95% CI = 1.02 to 1.88; CC + CT *vs*. TT: OR = 1.36, 95% CI = 1.10 to 1.69; CC *vs*. TT + CT: OR = 1.43, 95% CI = 1.13 to 1.81) and ESCC (C *vs*. T: OR = 1.17, 95% CI = 1.04 to 1.32; CC + CT *vs*. TT: OR = 1.28, 95% CI = 1.08 to 1.52).

**Conclusions:**

This meta-analysis provides the evidence that CYP1A1 MspI polymorphism may contribute to the EC development in the Chinese population.

## Review

### Background

Esophageal cancer (EC) is the eighth most common cancer and sixth most deadly cancer worldwide, with an estimated 482,300 new esophageal cancer cases and 406,800 deaths in 2008 [1]. Its incidence rates vary internationally; China and southern and eastern Africa are the relatively high risk areas [1]. In China, more strikingly, esophageal cancer ranks the fifth most common diagnosed cancer and fourth leading cause of cancer-related mortality [2]. The mechanisms of esophageal carcinogenesis have not been fully illustrated. Tobacco use [3,4], alcohol consumption [3,5], low socioeconomic status, poor oral hygiene, and nutritional deficiencies [6-8] have been identified as risk factors for esophageal cancer. Yet, only a subset of individuals exposed to these risk factors eventually develop esophageal cancer, indicating a pivotal role of genetic factors in the esophageal carcinogenesis. In recent years, several common low-penetrance genes have been identified as potential esophageal cancer susceptibility genes. An important one is cytochrome P450 1A1 (CYP1A1), which plays an essential role in the metabolic activation of major classes of tobacco procarcinogen such as aromatic amines and polycyclic aromatic hydrocarbons (PAHs). So it may affect the metabolism of the environmental carcinogens and alter susceptibility to esophageal cancer.

CYP1A1 enzyme is a member of the CYP superfamily and prone to mutation [9]. Agundez [9] revealed an association between CYP1A1 enzyme activity and the risk of developing several types of cancers, including EC. CYP1A1 A2455G and T3801C are two most commonly studied polymorphism loci. CYP1A1 T3801C polymorphism (MspI, rs4646903), also known as the m1 allele, is a substitution of T with C in the non-coding 3′-flanking region which resulting in a highly inducible activity of the enzyme [10,11]. The first research of the association between CYP1A1 MspI polymorphism and EC was reported by Hori and co-workers in 1997 among the Japan population [12]. As a consequence, many studies analyzed the influence of CYP1A1 MspI polymorphism on EC risk; however, no clear consensus was reached. Meta-analyses of studies on the gene in other ethnic groups have been reported elsewhere and produced conflicting results [13-15]. In order to lessen the impact of different genetic background, we performed this meta-analysis to assess the relationship of CYP1A1 MspI polymorphism with risk of EC in Chinese population.

## Methods

### Literature search

A comprehensive literature search was performed using the PubMed, Springer Link, Ovid, Chinese Wanfang Data Knowledge Service Platform, Chinese National Knowledge Infrastructure (CNKI), and Chinese Biology Medicine (CBM) for relevant articles published with the following Mesh terms: (‘Esophageal Neoplasms’ [MeSH] or ‘esophageal cancer’ or ‘esophageal tumor’ or ‘esophageal carcinoma’ or ‘esophageal squamous cell’ or ‘esophageal adenocarcinoma’) and (‘P4501A1’ or ‘CYP1A1’) and (China or Chinese or Taiwan). An upper date limit of 28 December 2014 was applied, and no lower date limit was used. The search was performed without any restrictions on language and focused on studies conducted in humans. Concurrently, the reference lists of reviews and retrieved articles were searched manually.

### Inclusion/exclusion criteria

For inclusion, the studies must have met the following criteria: (1) case–control study or cohort study studying on association between the CYP1A1 MspI polymorphisms and EC susceptibility; (2) all patients with the diagnosis of EC confirmed by pathological or histological examination; (3) sufficient published data about sample size, odds ratio (OR), and their 95% confidence interval (CI); (4) all participants were Chinese; (5) containing complete information about all genotype frequencies. Studies were excluded when they were: (1) not case–control study or cohort study; (2) duplicate of previous publication; (3) based on incomplete data; (4) meta-analyses, letters, reviews, or editorial articles.

### Data extraction

Information was extracted carefully from all eligible studies independently by two investigators according to the inclusion criteria listed above. Disagreements were resolved by discussion. The title and abstract of all potentially relevant articles were screened to determine their relevance. Full articles were also scrutinized if the title and abstract were ambiguous. The following data was collected from each study: first author’s surname, year of publication, geographical location, histopathology type, source of control, total numbers of cases and controls, and the numbers of cases and controls who harbored the MspI genotypes. If data from any category were not reported in the primary study, the items were designated ‘not stated’. We did not contact the author of the primary study to request the information.

### Statistical analysis

Crude odds ratios (ORs) together with their corresponding 95% CIs were used to assess the strength of association between the CYP1A1 MspI polymorphism and the risk of EC. The pooled ORs were performed for allele model (C *vs*. T), dominant model (CT + CC *vs*. TT), recessive model (CC *vs*. CT + TT), heterozygous model (CC *vs*. CT), and homozygous model (CC *vs*. TT), respectively. Between-study heterogeneity was assessed by the Q-statistics with *P* values <0.1. Dependent on the results of heterogeneity test among individual studies, the fixed effect model (Mantel-Haenszel) or random effect model (DerSimonian and Laird) was selected to summarize the combined OR and their 95% CI. Hardy-Weinberg equilibrium (HWE) was calculated by using the goodness-of-fit test, and deviation was considered when *P* < 0.05. The significance of the pooled OR was determined by the z test. Sensitivity analysis was conducted to verify stability of the meta-analysis using both models (the fixed effect model and random effect model). Begg’s funnel plots and Egger’s linear regression test were used to assess publication bias. In addition to the comparison among all subjects, we also performed stratification analyses by geographical locations, histopathology type, and source of control. All the statistical analysis was conducted by using STATA statistical package (version 10, STATA, College Station, TX, USA).

## Results

### Eligible studies

**Figure 1:**
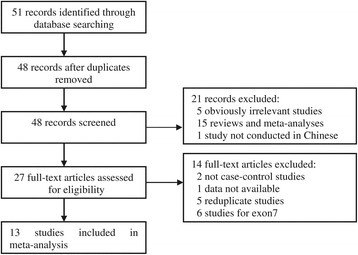
**Flow diagram of the literature search.**

Figure 1 graphically illustrates the trial flow chart. A total of 51 articles regarding CYP1A1 MspI polymorphism with respect to EC were identified. After screening the titles and abstracts, 24 articles were excluded because they were review articles, duplicates, not Chinese population, or irrelevant to the current study. In addition, of these published articles, 14 articles were excluded for full-text articles assessing due to case reports, reduplicate studies, or other polymorphisms of CYP1A1. Finally, 13 studies [16-28] including 1,519 EC cases and 1,962 controls were involved in this meta-analysis according to the inclusion criteria. The publication year of studies ranged from 2002 to 2014. Eleven of these studies were written in Chinese, two studies in English. The characteristics of the included studies are summarized in Table 1.

**Table 1:** **Characteristics of studies included in the meta-analysis**

**Reference**	**Source of controls**	**Geographical locations**	**Histopathology type**	**Case number**	**Control number**	**Case**			**Control**			**HWE**	
						**TT**	**CT**	**CC**	**TT**	**CT**	**CC**	**χ** ^**2**^	***P***
Wu 2002 [16]	HB	Taiwan	ESCC	146	344	60	65	21	136	146	62	4.16	0.041
Li 2002 [17]	HB	Shaxi	NS	73	75	20	30	23	37	25	13	4.96	0.026
Yin 2005 [18]	HB	Jiangsu	ESCC	106	106	42	54	10	41	49	16	0.05	0.829
Zhu 2005 [19]	HB	Shaxi	ESCC + EAC	163	166	43	75	45	75	67	24	1.96	0.162
Han 2005 [20]	HB	Shaxi	ESCC	89	98	25	39	25	47	38	13	1.37	0.241
Lu 2006 [21]	PB	Xinjiang	ESCC	64	116	23	28	13	44	56	16	0.07	0.786
Yin 2010 [22]	HB	Xinjiang	NS	96	174	16	45	35	17	88	69	2.13	0.144
Ji 2010 [23]	PB	Gansu	ESCC	189	216	49	95	45	70	98	48	1.49	0.222
Gao 2012 [24]	PB	Ningxia	ESCC	40	80	15	17	8	28	41	11	0.43	0.511
Huang 2012 [25]	PB	Guangxi	ESCC	98	100	38	41	19	40	43	17	0.85	0.358
Zhang 2013 [26]	PB	Shandong	ESCC	138	170	34	78	26	59	83	28	0.02	0.896
Yun 2014 [27]	PB	Henan	ESCC	157	157	73	72	12	95	50	12	2.11	0.146
Zhang 2011 [28]	PB	Henan	ESCC + EAC	160	160	97		63	128		32	-	

PB population-based, HB hospital-based, ESCC esophageal squamous cell carcinoma, EAC esophageal adenocarcinoma, NS not stated.

### Meta-analysis results

**Figure 2:**
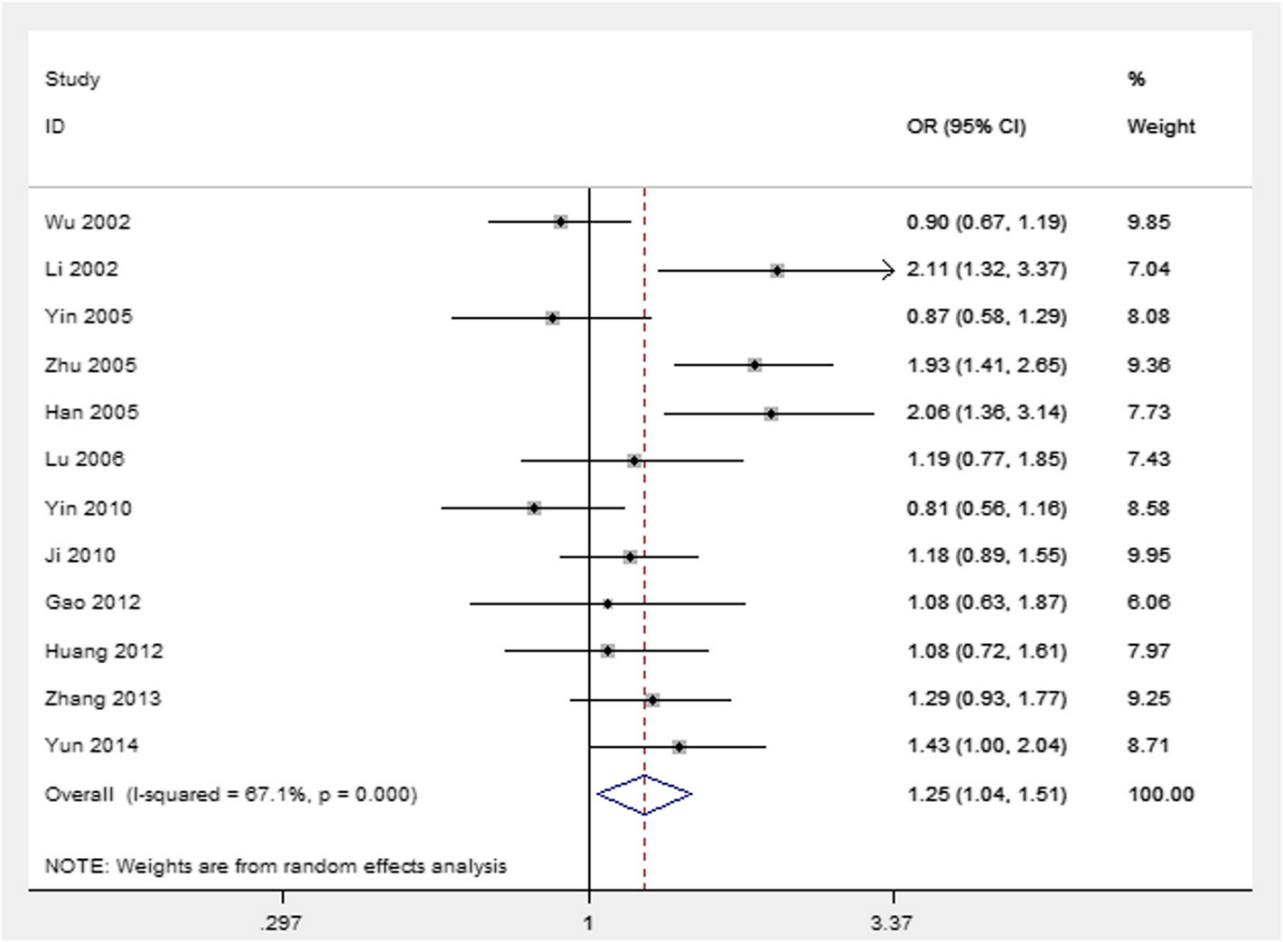
**Forest plot (random-effect model) of lung cancer risk associated with CYP1A1 MspI polymorphism using the allele genetic model.**

Table 2 lists the primary results. Overall, a significantly elevated risk of EC was associated with two variants of CYP1A1 MspI (for CC and CT combined *vs*. TT: OR = 1.35, 95% CI = 1.06 to 1.72, *P* = 0.005 for heterogeneity; for CC *vs*. TT and CT: OR = 1.35, 95% CI = 1.03 to 1.76, *P* = 0.008 for heterogeneity). For the allele C *vs*. allele T, the pooled OR was 1.25 (95% CI = 1.04 to 1.51; *P* = 0.000 for heterogeneity) (Figure 2). However, there was significant heterogeneity between studies. Hence, we then performed subgroup analysis by geographical locations, histopathology type, and source of control. In the stratified analysis by geographical locations, significantly increased risks were found in the population from North China (C *vs*. T: OR = 1.38, 95% CI = 1.12 to 1.70; CC *vs*. TT: OR = 1.72, 95% CI = 1.16 to 2.56; CC + CT *vs*. TT: OR = 1.52, 95% CI = 1.14 to 2.02; CC *vs*. TT + CT: OR = 1.55, 95% CI = 1.17 to 2.06) but not found in the South. In the stratified analysis by source of controls, significantly increased risks were found in the population-based studies (C *vs*. T: OR = 1.22, 95% CI = 1.05 to 1.42; CC *vs*. TT: OR = 1.38, 95% CI = 1.02 to 1.88; CC + CT *vs*. TT: OR = 1.36, 95% CI = 1.10 to 1.69; CC *vs*. TT + CT: OR = 1.43, 95% CI = 1.13 to 1.81) but not found in the hospital-based studies. In the subgroup analysis by histopathology type, significantly increased association was found in esophageal squamous cell carcinoma (ESCC) (C *vs*. T: OR = 1.17, 95% CI = 1.04 to 1.32; CC + CT *vs*. TT: OR = 1.28, 95% CI = 1.08 to 1.52).

**Table 2:** **Main results in the total and subgroup analysis**

**Analysis model**	**Study groups**	***n***	**Random-effect model**	**Fixed-effect model**	**Heterogeneity**	
			**OR (95% CI)**	**OR (95% CI)**	**χ** ^**2**^	***P***
C *vs*. T	Total analysis	12	*1.25* (*1.04 to 1.51*)	*1.23* (*1.11 to 1.37*)	33.46	0.000
	PB	6	*1.22* (*1.05 to 1.42*)	*1.22* (*1.05 to 1.42*)	1.49	0.914
	HB	6	1.30 (0.90 to 1.90)	1.25 (1.08 to 1.44)	31.93	0.000
	ESCC	9	*1.18* (*1.00 to 1.39*)	*1.17* (*1.04 to 1.32*)	14.48	0.070
	South China	3	0.93 (0.76 to 1.14)	0.93 (0.76 to 1.14)	0.71	0.702
	North China	9	*1.38* (*1.12 to 1.70*)	*1.37* (*1.21 to 1.55*)	22.21	0.005
CC *vs*. TT	Total analysis	12	1.42 (0.99 to 2.04)	1.42 (1.15 to 1.75)	29.24	0.002
	PB	6	*1.38* (*1.02 to 1.88*)	*1.38* (*1.02 to 1.88*)	0.45	0.994
	HB	6	1.46 (0.70 to 3.04)	1.45 (1.09 to 1.93)	28.75	0.000
	ESCC	9	1.29 (0.94 to 1.77)	1.27 (1.00 to 1.62)	12.34	0.137
	South China	3	0.82 (0.54 to 1.25)	0.82 (0.54 to 1.24)	1.26	0.531
	North China	9	*1.72* (*1.16 to 2.56*)	*1.73* (*1.35 to 2.22*)	18.97	0.015
CC *vs*. CT	Total analysis	12	1.09 (0.89 to 1.33)	1.09 (0.89 to 1.32)	10.90	0.452
	PB	6	1.07 (0.80 to 1.43)	1.06 (0.80 to 1.42)	2.93	0.711
	HB	6	1.12 (0.78 to 1.59)	1.11 (0.85 to 1.45)	7.94	0.160
	ESCC	9	1.01 (0.80 to 1.28)	1.01 (0.80 to 1.28)	7.91	0.442
	South China	3	0.80 (0.53 to 1.21)	0.80 (0.53 to 1.21)	1.53	0.464
	North China	9	1.19 (0.95 to 1.50)	1.19 (0.95 to 1.50)	6.63	0.577
CC + CT *vs*. TT	Total analysis	12	*1.35* (*1.06 to 1.72*)	*1.37* (*1.17 to 1.59*)	26.54	0.005
	PB	6	*1.36* (*1.10 to 1.69*)	*1.36* (*1.10 to 1.69*)	4.10	0.536
	HB	6	1.39 (0.86 to 2.23)	1.37 (1.11 to 1.70)	22.44	0.000
	ESCC	9	*1.28* (*1.04 to 1.59*)	*1.28* (*1.08 to 1.52*)	11.73	0.164
	South China	3	0.97 (0.73 to 1.28)	0.97 (0.73 to 1.28)	0.11	0.947
	North China	9	*1.52* (*1.14 to 2.02*)	*1.58* (*1.32 to 1.89*)	18.30	0.019
CC *vs*. TT + CT	Total analysis	13	*1.35* (*1.03 to 1.76*)	*1.34* (*1.13 to 1.59*)	26.94	0.008
	PB	7	*1.41* (*1.06 to 1.89*)	*1.43* (*1.13 to 1.81*)	8.26	0.219
	HB	6	1.30 (0.79 to 2.14)	1.25 (0.98 to 1.60)	18.16	0.003
	ESCC	9	1.14 (0.88 to 1.47)	1.12 (0.90 to 1.40)	10.22	0.250
	South China	3	0.82 (0.56 to 1.20)	0.81 (0.56 to 1.19)	1.63	0.443
	North China	10	*1.55* (*1.17 to 2.06*)	*1.53* (*1.26 to 1.86*)	17.20	0.046

PB population-based, HB hospital-based, ESCC esophageal squamous cell carcinoma, South China including Taiwan, Jiangsu, and Guangxi; North China including Shaxi, Xinjiang, Gansu, Ningxia, Shandong, and Henan.

### Sensitive analysis and bias diagnosis

**Figure 3:**
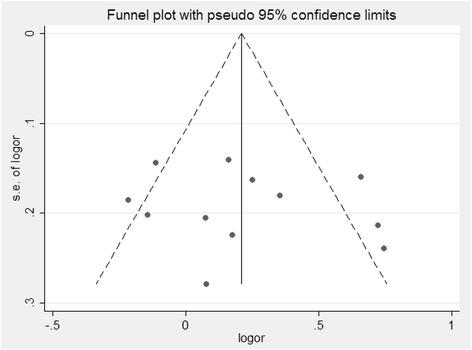
**Begg’s funnel plot of CYP1A1 MspI polymorphism and lung cancer risk under the allele genetic model.**

In order to compare the difference and evaluate the sensitivity of the meta-analyses, we used both models (the fixed effect model and random effect model) to evaluate the stability of the meta-analysis. All the significant results were not materially altered (Table 2). Hence, results of the sensitivity analysis suggest that the data in this meta-analysis are relatively stable and credible. The Begg’s funnel plot and Egger’s test were performed to assess the publication bias of literatures. The shape of the funnel plots did not reveal obvious asymmetry (Figure 3). Then, the Egger’s test was used to provide statistical evidence of funnel plot symmetry. The Egger’s test indicated that there were no obvious publication bias under the allele model in overall analyses (t = −0.62, *P* = 0.550).

## Discussion

CYP genes are large families of endoplasmic and cytosolic enzymes that catalyze the activation and detoxification, respectively, of reactive electrophilic compounds, including many environmental carcinogens (for example, benzo [a] pyrene). CYP1A1 is a phase I enzyme that regulates the metabolic activation of major classes of tobacco procarcinogens, such as aromatic amines and PAHs [29]. Thus, CYP1A1 may affect the metabolism of environmental carcinogens and alter the susceptibility to cancers, including EC. Although many studies analyzing the research results about the association between CYP1A1 MspI polymorphism and EC, definite conclusions cannot be drawn [13-15]. Therefore, we did this meta-analysis to estimate the relationship between CYP1A1 MspI polymorphism and susceptibility to EC among the Chinese population, in order to lessen the impact of different genetic background. This meta-analysis involved 13 articles with 1,519 EC cases and 1,962 controls. The results indicated a significant association between CYP1A1 MspI gene polymorphism and EC risk in the Chinese population. The sensitivity analysis confirmed the reliability and stability of the meta-analysis, and no publication bias was found among studies by Egger’s test. Therefore, the findings from our meta-analysis provide a strong evidence for the association between CYP1A1 MspI polymorphism and risk of EC in the Chinese population. Our results were inconsistent with a previously published meta-analysis in Chinese, which indicated that CYP1A1 Ile/Val genetic polymorphisms, but not CYP1A1 MspI polymorphisms, are associated with an increased digestive tract cancers risk in Chinese populations [30]. It was also inconsistent with the findings for Caucasians [13-15,31]. The exact mechanism for the ethnic discrepancy is uncertain but differences in underlying genetic backgrounds and social factors among different populations studied may be important. Ethnically diverse subjects may have unique cultures and lifestyles that can contribute to different genetic characteristics and susceptibility to specific cancers. Also, this inconsistency may be due to the smaller sample sizes for Chinese used in previous meta-analyses.

When we performed stratified analyses by geographical locations, histopathology type, and source of control, significant association with susceptibility for the development of EC was found in North China, in ESCC, and population-based studies; however, it was not found in South China and hospital-based studies. These can be influenced by some factors. First, the hospital-based studies usually have some biases because such controls may just represent a sample of ill-defined reference population and may not be representative of the general population. Second, genetic risk factors for EC in North and South China are different [32]. Last but not the least, for the relevant small sample size, only three studies from South China was included in the meta-analysis. With regard to heterogeneity, some of the factors extracted in this study were the main source of heterogeneity. But it might also make attributions for other unknown factors, such as dietary habits, dinking status, other environmental exposures (passive smoking and cooking oil fume), family history of cancer, other genetic-related respiratory diseases as well as other related genetic polymorphisms.

The pathways of carcinogen metabolism are complex, mediated by the activity of multiple enzymes. The effect of any single gene might have a limited impact on EC risk than have so far been anticipated. EC has some known major environmental determinants other than tobacco smoke, and large studies with detailed exposure information are needed to evaluate reliably any moderate genetic effects. Otherwise, some limitations should be acknowledged. Firstly, we did not perform subgroup analysis on smoking status because of the lack of sufficient data. Another potential limitation was that our results were based on unadjusted estimates. More precise analyses can be conducted if individual data were available, which would allow for the adjustment by other covariates including age, sex, race, and other factors. Finally, heterogeneity can interfere with the interpretation of the results of a meta-analysis. Although we minimized this likelihood by performing a careful search of published studies and subgroup analyses, significant inter-study heterogeneity nevertheless existed in the comparison of geographical locations. In spite of these limitations, our meta-analysis still had some advantages. We obeyed the inclusion and exclusion criteria strictly to reduce selection bias. A funnel plot and Egger’s linear regression test was used to assess publication bias. In addition, the impact of different genetic background was lessened by means of including the studies performed in the Chinese population only, and the test of the Hardy-Weinberg equilibrium for distribution of the genotypes in control groups suggested that there was almost no significantly different genetic background among the participants.

## Conclusions

In conclusion, our meta-analysis supports that CYP1A1 MspI polymorphism might contribute to individual susceptibility to EC in the Chinese population. Concerning EC with multifactorial etiology, to further evaluate gene-gene and gene-environment interactions on CYP1A1 MspI polymorphism and EC, larger studies in selected populations with different environmental background or other risk factors are required. Such studies may eventually lead to have a better, comprehensive understanding of the association between the CYP1A1 MspI polymorphism and EC risk.
